# Geometric distortion assessment in 3T MR images used for treatment planning in cranial Stereotactic Radiosurgery and Radiotherapy

**DOI:** 10.1371/journal.pone.0268925

**Published:** 2022-05-23

**Authors:** Stefanos Theocharis, Eleftherios P. Pappas, Ioannis Seimenis, Panagiotis Kouris, Dimitrios Dellios, Georgios Kollias, Pantelis Karaiskos

**Affiliations:** 1 Medical Physics Laboratory, Medical School, National and Kapodistrian University of Athens, Athens, Greece; 2 Medical Physics and Gamma Knife Department, Hygeia Hospital, Marousi, Greece; St. Vincent Medical Center, UNITED STATES

## Abstract

Magnetic Resonance images (MRIs) are employed in brain Stereotactic Radiosurgery and Radiotherapy (SRS/SRT) for target and/or critical organ localization and delineation. However, MRIs are inherently distorted, which also impacts the accuracy of the Magnetic Resonance Imaging/Computed Tomography (MRI/CT) co-registration process. In this phantom-based study, geometric distortion is assessed in 3T T2-weighted images (T2WIs), while the efficacy of an MRI distortion correction technique is also evaluated. A homogeneous polymer gel-filled phantom was CT-imaged before being irradiated with 26 4-mm Gamma Knife shots at predefined locations (reference control points). The irradiated phantom was MRI-scanned at 3T, implementing a T2-weighted protocol suitable for SRS/SRT treatment planning. The centers of mass of all shots were identified in the 3D image space by implementing an iterative localization algorithm and served as the evaluated control points for MRI distortion detection. MRIs and CT images were spatially co-registered using a mutual information algorithm. The inverse transformation matrix was applied to the reference control points and compared with the corresponding MRI-identified ones to evaluate the overall spatial accuracy of the MRI/CT dataset. The mean image distortion correction technique was implemented, and resulting MRI-corrected control points were compared against the corresponding reference ones. For the scanning parameters used, increased MRI distortion (>1mm) was detected at areas distant from the MRI isocenter (>5cm), while median radial distortion was 0.76mm. Detected offsets were slightly higher for the MRI/CT dataset (0.92mm median distortion). The mean image distortion correction improves geometric accuracy, but residual distortion cannot be considered negligible (0.51mm median distortion). For all three datasets studied, a statistically significant positive correlation between detected spatial offsets and their distance from the MRI isocenter was revealed. This work contributes towards the wider adoption of 3T imaging in SRS/SRT treatment planning. The presented methodology can be employed in commissioning and quality assurance programmes of corresponding treatment workflows.

## 1. Introduction

Cranial Stereotactic Radiosurgery and Radiotherapy (SRS/SRT) are commonly employed for the management of small lesions in the brain related to either benign (e.g., meningiomas and acoustic neuromas), malignant (e.g., metastases) or functional (trigeminal neuralgia) disorders [1–7]. In any case, it is paramount that radiation dose is very accurately delivered to the patient to increase lesion control probability while minimizing the risk for radiation-induced toxicity to the surrounding normal parenchyma and critical brain structures. This requirement is associated with the dosimetric characteristics of SRS/SRT i.e., the high doses per fraction considered (typically 6-24 Gy or even 60 Gy for functional disorders), as well as the conformal dose distributions and rapid dose fall-off (dose gradient) employed [8]. Moreover, the spatial margins applied to the Gross Tumor Volume (GTV) to ensure target coverage are minimal (typically 1-2 mm), while a zero-margin approach is not uncommon [9–11]. Consequently, spatial inaccuracies of the order of 1 mm can result in considerable target underdosage and/or increased risk for radiation-induced toxicity [12–14].

Magnetic Resonance Imaging (MRI) offers superior soft-tissue contrast [as compared to Computed Tomography (CT)], especially following intravenous contrast agent injection [15]. Therefore, MRI is the modality of choice for cranial SRS/SRT treatment planning. Both T1- and T2-weighted images (T1WIs and T2WIs, respectively) images are commonly employed for target identification, localization and delineation and/or precise contouring of adjacent critical structures in the brain [6, 7, 16]. However, this comes at the expense of reduced spatial fidelity, since Magnetic Resonance images (MRIs) are inherently distorted [17]. Sources of geometric distortion are either machine-related (B0 inhomogeneity and gradient field non-linearity) or patient-induced (susceptibility differences and the chemical shift effect) [17–20]. In both cases, a spatial mis-encoding is introduced to the read-out signal, resulting in a geometric offset of the pixel’s representation in the image space. For a given offset to the reference Larmor resonance frequency, the magnitude of distortion is inversely proportional to the magnetic field gradient strength or, equivalently, the receiver bandwidth per pixel. On the other hand, increasing the receiver bandwidth will result in reduced signal-to-noise ratio (SNR) in the image, potentially obscuring tiny brain lesions or reducing the sharpness of their boundaries [17, 18, 21, 22].

To enhance lesion conspicuity, there is a recent trend for using high field strength (i.e., 3T) MRI systems in SRS/SRT treatment planning, also facilitated by their increasing availability in clinical practice. Their main advantage is the increase in SNR for the same scanning time. Alternatively, 3T systems can enable imaging in higher spatial resolution (<1mm^3^), at no considerable cost in scanning time. However, the distortion magnitude associated with susceptibility and chemical shift phenomena also greatly increases for the same imaging parameters [22–24]. Moreover, machine-related distortions (i.e., B0 inhomogeneity and gradient field non-linearities) are also expected to be larger [12]. Therefore, 1.5T MRI is still the modality of choice in cranial SRS/SRT treatment planning [25].

In addition to directly introducing spatial inaccuracy in target and critical organ localization and delineation, MRI-induced distortion might also compromise the accuracy of the MRI/CT spatial co-registration step [26, 27], commonly performed in CT-based SRS/SRT treatment workflows. Thus, it is crucial that distortion levels are minimized in order not to risk compromising the treatment efficiency. Apart from employing vendor-supplied methods for distortion reduction, several approaches have been proposed for post-imaging distortion correction [19, 28–32]. The majority of these studies are based on the reversed read gradient method [33] or employ the field mapping technique [34]. However, the literature has mainly focused on 1.5T MRI, while the efficacy of the correction schemes has been investigated only for T1WIs. Recognising the need to address the issues related to the use of MRI data in treatment planning and stereotactic procedures, the American Association of Physicists in Medicine (AAPM) has formed TG-117 (final report is pending).

Studies reporting on MRI-related distortion levels in images acquired at 3T are quite scarce. Most of these studies focus on extracranial and/or conventional radiotherapy applications, while only T1WIs have been considered [12, 18, 19, 24, 35]. However, cranial SRS/SRT treatment planning procedures entail more stringent spatial tolerances [22], while T2WIs may well be employed. This phantom-based work focuses specifically on cranial SRS/SRT applications and investigates, over the whole volume of interest, the spatial fidelity of T2w images acquired at 3T with a clinically used protocol. The impact of distortion on the MRI/CT registration accuracy is also addressed. In addition, the mean image distortion correction approach [36] is implemented and evaluated in 3T images for the first time.

## 2. Materials and methods

### 2.1 Phantom and image acquisitions

A custom-made phantom, described in a previous study [37] for distortion detection in a 1.5T system, was also used herein. Briefly, a spherical hollow container (diameter of 16 cm), made of acrylic, was filled with homogeneous gelatin-based polymer gel dosimeter (physical density of 1.031 g/cc) of the formulation referred to as VIP [38].

The phantom was CT-scanned at 120 kVp with acquisition parameters given in Table 1. Images were imported to the Leksell GammaPlan (ELEKTA AB, Stockholm, Sweden) treatment planning system (TPS). A plan comprising 26 Gamma Knife shots (GK, ELEKTA AB, Stockholm, Sweden), prescribed at 26 predefined locations was prepared. For each shot, all 8 sectors were aligned with the 4-mm collimator. It is noted that the 4-mm shots deliver dose distributions of spherical shape with no apparent dose plateau [39]. Treatment delivery was performed using a GK Perfexion (ELEKTA AB, Stockholm, Sweden) irradiation unit and the Leksell stereotactic frame mounted on the phantom.

**Table 1:** CT and MRI acquisition parameters for the scans performed in this study.

**CT scanner**	**Scanning Mode**	**Voxel size (mm3)**	**Matrix size**	**kVp (kV)**		
Siemens Volume	Helical	0.43 × 0.43 × 1	512 × 512	120		
**MRI scanner**	**Pulse sequence**	**Voxel size (mm^3^)**	**Matrix size**	**Bandwidth (Hz/pixel)**	**TE/TR/FA (ms/ms/degrees)**	**Read gradient direction**
Philips Achieva 3T	3D T2w TSE	1 × 1 × 1	256 × 256	584	140/2700/90	A– P (forward scan)
Philips Achieva 3T	3D T2w TSE	1 × 1 × 1	256 × 256	584	140/2700/90	P– A (reverse scan)

Abbreviations: T2w: T2-weighted; TSE: Turbo Spine Echo; TE: Echo Time; TR: Repetition Time; FA: Flip Angle; A: Anterior; P: Posterior.

After irradiation, the phantom was scanned at 3T (Achieva, Philips Medical Systems, Eindhoven, The Netherlands) with a clinical T2-weighted protocol (scanning parameters are given in Table 1). To implement the mean image distortion correction technique [36], two MRI scans were performed with identical imaging parameters, except for a reversal of the polarity of the frequency encoding gradient. More specifically, the readout gradient was initially set on the Anterior-Posterior direction and then changed to Posterior-Anterior, assuming the standard Head-First-Supine (HFS) position for the phantom. Hereinafter, the former will be referred to as the forward scan while the latter as the reverse scan. It is noted that the forward images are used in clinical SRS/SRT treatment planning only by convention, and there is no limitation in using the reverse ones, instead.

### 2.2 Control point localization algorithm

**Fig 1:**
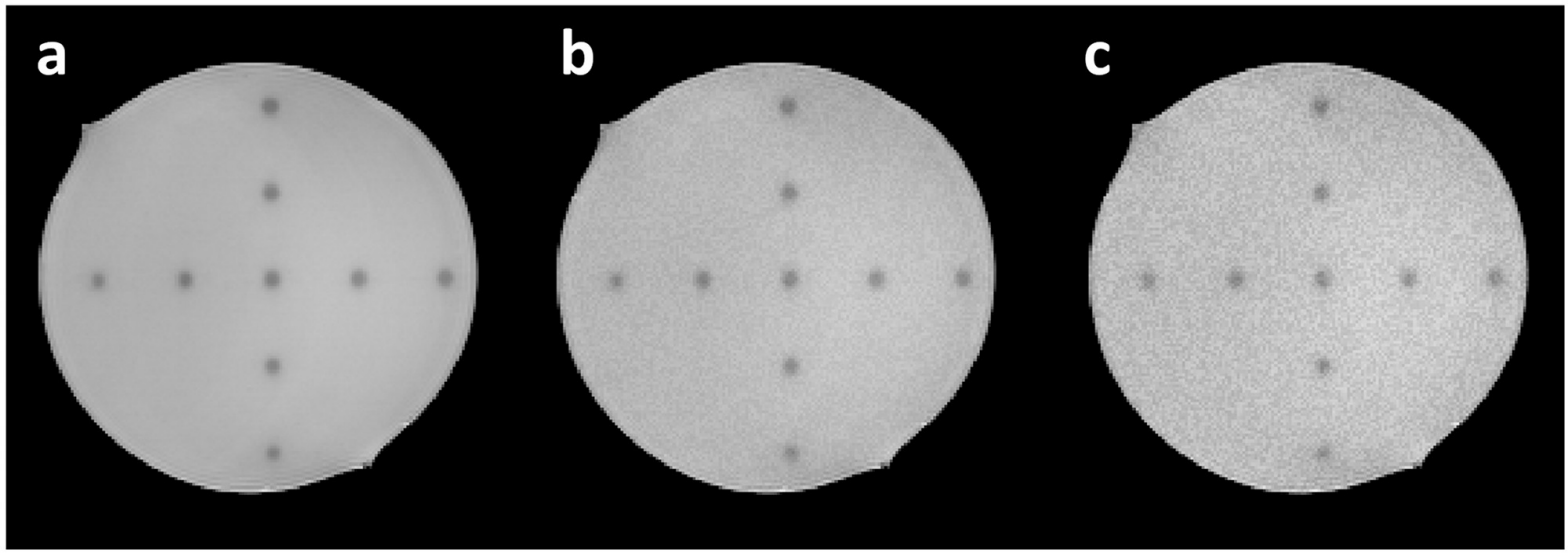
MRIs of the irradiated phantom. (a) An indicative axial image of the irradiated phantom, depicting the polymerized areas (lower T2-weighted signal) as a result of GK 4-mm shot delivery. (b) and (c) The same slice after applying 5% and 10% random noise, respectively, for estimating control point localization uncertainty.

Acquired T2WIs exhibited adequate contrast to highlight radiation-induced polymerization at high dose areas, as shown in Fig 1a for an indicative slice. Thus, a virtual grid of control points (at known predefined locations) was introduced within the homogeneous phantom. All acquired images were imported to MATLAB R2019a (The MathWorks, Inc, MI) for analysis. In all images and for all 26 GK shots, the center of mass of the polymerized (high dose) area was calculated by implementing an iterative calculation algorithm, described elsewhere [37]. In short, the geometric centroid of a polymerized volume is determined in 3D by applying a seed signal threshold locally and, using standard MATLAB routines, the image is transformed into a binary one and the corresponding centroid is calculated. The process is repeated after changing the applied threshold (by a user-selected step), resulting into a new estimation of the centroid. The control point location (corresponding to one of the 26 shots) within the image coordinate system is determined, independently for each axis, by calculating the average location for all applied threshold levels.

The TPS allows for exporting the dose distribution in either the MRI or the CT Digital Imaging and Communications in Medicine (DICOM) coordinate system, for the same set of shots defined in the plan, depending on the imported image stack used for treatment planning (primary image volume). To calculate the reference control point locations in each DICOM coordinate system, the dose distribution resulting from all 26 shots was exported from the TPS in DICOM-RT file format. The methodology described above was applied to determine the center of mass of the high dose area, for each shot, yielding the corresponding reference control point location.

The reference dose distribution in the MRI DICOM coordinate system was used in sections 2.3 and 2.5 (not involving CT images). The reference dose distribution in the CT DICOM coordinate system was used for the purposes of section 2.4 (involving the MRI/CT registered dataset), although final comparisons were performed in the MRI DICOM coordinate system as explained below. Calculated coordinates of the reference control points in the MRI DICOM coordinate system are provided in Table 2.

**Table 2:** Reference control point locations (i.e., centers of the GK shots) in the MRI DICOM coordinate system.

Shot ID	X coordinate (mm)	Y coordinate (mm)	Z coordinate (mm)	Distance from the MRI isocenter (mm)
1	-31.4	-21.7	57.7	69.2
2	-1.4	-46.7	56.6	73.4
3	23.6	-21.7	57.3	65.7
4	-1.4	3.3	58.3	58.4
5	-1.4	-21.7	57.5	61.5
6	-51.6	-20.7	27.9	62.2
7	-1.7	-70.7	25.8	75.3
8	48.4	-20.7	27.1	59.2
9	-1.6	19.3	28.8	34.7
10	-1.6	-20.7	27.5	34.5
11	-31.9	-19.7	-2.3	37.6
12	-61.9	-19.7	-2.0	65.0
13	-1.9	-49.7	-3.5	49.9
14	-2.0	-79.7	-4.4	79.8
15	28.1	-19.8	-2.8	34.5
16	58.1	-19.8	-3.0	61.5
17	-1.9	10.2	-1.6	10.5
18	-1.8	40.2	-0.6	40.2
19	-1.9	-19.8	-2.5	20.0
20	-52.2	-18.7	-32.1	64.1
21	-2.2	-68.8	-34.1	76.8
22	47.9	-18.8	-32.9	61.1
23	-2.1	31.2	-30.9	44.0
24	-2.2	-18.8	-32.5	37.6
25	22.7	7.2	-61.5	65.9
26	-2.4	-17.8	-61.8	64.4

The software tool described above has been shown to estimate the center of mass related to each shot with an accuracy of 0.1 mm [37]. However, assuming that noise levels are not the same throughout the image volume, a simple test was developed to further assess the accuracy of the control point localization algorithm in the presence of increased noise in the MRIs. More specifically, using a random number generator (incorporated in MATLAB), noise was deliberately increased in the forward MRIs by 5% and 10%. Resultant images are shown in Fig 1b and 1c, respectively. The centroids of the polymerized areas in the artificially noisy images were calculated using the same algorithm and obtained results were compared with the corresponding locations obtained from the original (less noisy) image (Fig 1a).

### 2.3 MRI overall distortion assessment

Using the methodology described, the overall MRI spatial distortion was calculated on each axis, *i*, by:

$$\begin{array}{cccc} d_{i}^{MRI} & =i^{MRI}-i^{RT}, & & \text{for}\;i=x,y,z\;\text{of}\;\text{the}\;\text{MRI}\;\text{DICOM}\;\text{coordinate}\;\text{system} \end{array}$$ (1)

where *i*^*MRI*^ and *i*^*RT*^ represent the control point location (i.e., center of mass) identified in the forward MRI scan and the reference TPS-calculated (RTDOSE) dose distribution, respectively. Consequently, the overall MRI distortion, $d_{R}^{MRI}$, was calculated as the radial distance between the reference and evaluated locations, i.e.:

$$\begin{array}{c} d_{R}^{MRI}=\sqrt{{\left(d_{x}^{MRI}\right)}^{2}+{\left(d_{y}^{MRI}\right)}^{2}+{\left(d_{z}^{MRI}\right)}^{2}} \end{array}$$ (2)

### 2.4 MRI/CT registered overall spatial accuracy

In CT-based SRS/SRT procedures, CT images are considered as the primary image stack used for treatment planning. To quantify the spatial accuracy of the MRI/CT co-registration step (potentially compromised due to MRI distortion), CT images of the unirradiated phantom and MRIs of the irradiated phantom were imported to Monaco TPS (ELEKTA AB, Stockholm, Sweden), simulating a linac-based SRS/SRT procedure. The mutual information algorithm, incorporated in the TPS, was used to spatially co-register MRI and CT coordinate systems. The rigid transformation matrix was calculated by the TPS after selecting a rectangular region of interest covering the entire phantom volume but excluding the stereotactic frame and the localization box. Result of the registration step was visually inspected and verified according to the recommendations of AAPM TG-132 [26]. The relevant rigid transformation matrix was exported from the TPS in DICOM format and imported to MATLAB.

After applying the inverse rigid transformation (i.e., CT to MRI) to the CT images, as well as to the corresponding RTDOSE dose distribution (i.e., the one in the CT DICOM coordinate system), all datasets were registered to the MRI DICOM coordinate system. The spatial offset between control points identified in the MRI/CT co-registered images and the corresponding reference control point locations were calculated, i.e.:

$$\begin{array}{cccc} d_{i}^{MRI/CT} & =i^{MRI/CT}-i^{RT}, & & \text{for}\;i=x,y,z\;\text{of}\;\text{the}\;\text{MRI}\;\text{DICOM}\;\text{coordinate}\;\text{system} \end{array}$$ (3)

Accordingly, the overall geometric offset, $d_{R}^{MRI/CT}$ was calculated according to:

$$\begin{array}{c} d_{R}^{MRI/CT}=\sqrt{{\left(d_{x}^{MRI/CT}\right)}^{2}+{\left(d_{y}^{MRI/CT}\right)}^{2}+{\left(d_{z}^{MRI/CT}\right)}^{2}} \end{array}$$ (4)

### 2.5 Efficacy of MRI distortion correction

The mean image distortion correction has been proposed [36] and validated in a number of studies [32, 36, 40] employing 1.5T MRI. The theoretical basis of this distortion correction method is the fact that reversing the read gradient polarity, distortion magnitude remains unaffected, while distortions associated with B0 inhomogeneity, susceptibility differences and the chemical shift effect (collectively referred to as sequence dependent distortions) change sign. The mean image approach is rather simple in concept: a new image is created by averaging the pixel intensity in the two corresponding opposite gradient polarity images, on a pixel-by-pixel basis. Effectively, sequence dependent distortions are minimized, while gradient field nonlinearity-induced ones remain unaffected.

Using both the forward and reverse read gradient polarity scans (Table 1), mean images were created in MATLAB. Subsequently, the control points were identified on the corrected images by implementing the same control point localization algorithm. Spatial offsets between MRI-corrected control points locations, $d_{i}^{MRIcorr}$, and corresponding reference ones were calculated, yielding the residual spatial distortion:

$$\begin{array}{cccc} d_{i}^{MRIcorr} & =i^{MRIcorr}-i^{RT}, & & \text{for}\;i=x,y,z\;\text{of}\;\text{the}\;\text{MRI}\;\text{DICOM}\;\text{coordinate}\;\text{system} \end{array}$$ (5)

$$\begin{array}{c} d_{R}^{MRIcorr}=\sqrt{{\left(d_{x}^{MRIcorr}\right)}^{2}+{\left(d_{y}^{MRIcorr}\right)}^{2}+{\left(d_{z}^{MRIcorr}\right)}^{2}} \end{array}$$ (6)

## 3. Results

### 3.1 Uncertainty estimation

Table 3 presents the results of the test performed to evaluate the effect of image noise on the uncertainty related to the control point localization algorithm. By adding random noise of 5% (shown in Fig 1b), a radial discrepancy of <0.1 mm was observed, suggesting that such noise levels cannot considerably affect the results. However, 10% noise addition resulted in a control point localization offset of 0.26 mm (Table 3). Throughout this study, the maximum detected radial discrepancy of 0.26 mm is adopted. Adding an inherent 0.1 mm uncertainty [37] of the developed software routines, results in a combined uncertainty in control point localization of 0.28 mm. However, distortion detection requires the localization of two respective control point locations (i.e., the reference and evaluated ones). Thus, a total combined uncertainty of 0.39 mm is ascribed to all results presented in the following sections.

**Table 3:** Sensitivity of the control point localization algorithm with respect to added noise in the images. Maximum discrepancies on each axis are given. The MRI DICOM coordinate system is adopted.

	Discrepancy with respect to original image			
	Δx(mm)	Δy(mm)	Δz(mm)	Δr(mm)
**5% added noise**	0.04	0.02	0.04	0.06
**10% added noise**	0.19	0.12	0.13	0.26

Abbreviation: $\Delta r=\sqrt{{\left(\Delta x\right)}^{2}+{\left(\Delta y\right)}^{2}{+\Delta z)}^{2}}$

### 3.2 MRI distortion assessment

**Fig 2:**
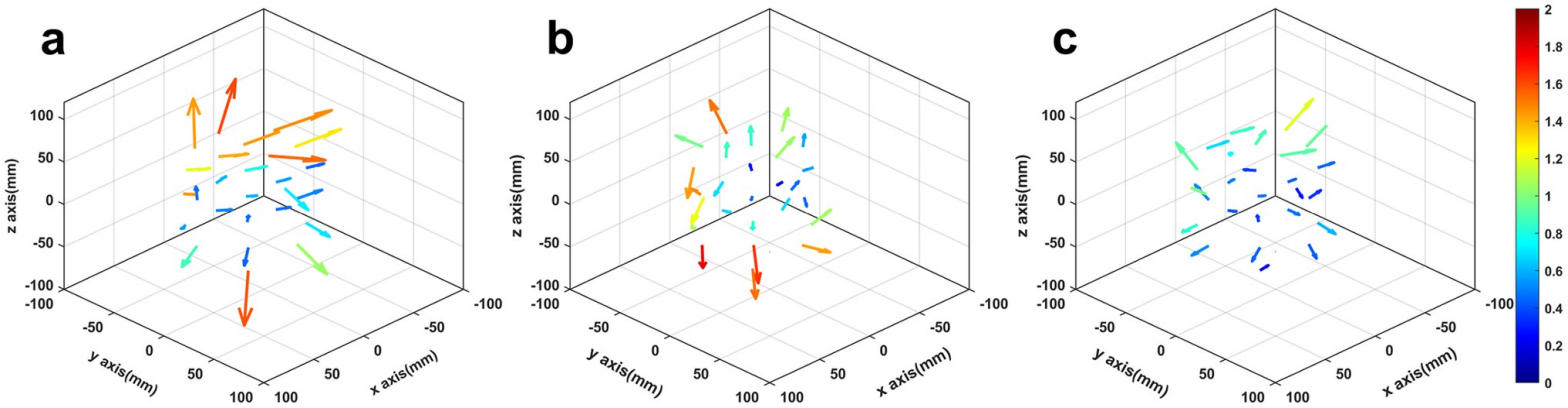
Vectors representing total spatial offset, *d*_*R*_. (a) MRI forward images, (b) MRI/CT dataset and (c) MRI-corrected images. Vectors’ origins correspond to the positions of the reference control points. Vectors’ lengths have been magnified to increase visibility but are proportional to the detected offsets which are quantified by the colorbar in mm. The MRI DICOM coordinate system is adopted.

**Fig 3:**
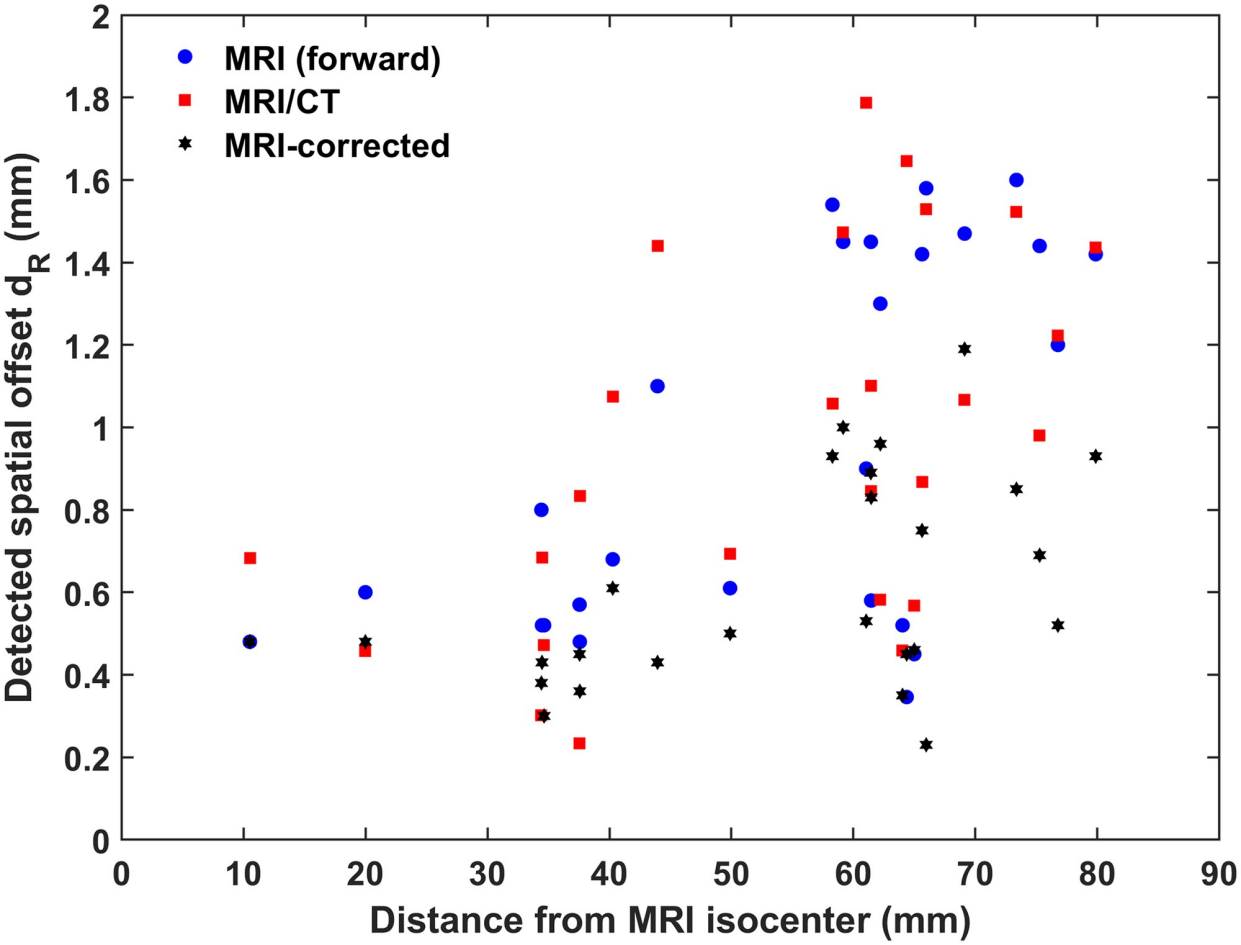
Magnitude of the radial spatial offset, |*d*_*R*_|, for all 26 control points with respect to their distance from the MRI isocenter. Using Spearman’s correlation coefficient, a statistically significant positive correlation between the distance to the MRI isocenter and detected radial offset was revealed for all three datasets.

MRI distortion vectors corresponding to the forward scan are presented for all 26 control point positions in Fig 2a. As expected, distortion levels close to the MRI isocenter (i.e., position (0,0,0) in the DICOM image space) are minimal. Distortion greatly increases towards the periphery of the mapped volume. This is more evident in Fig 3, where the overall distortion magnitude is presented against the distance of the control points from the MRI isocenter. The Spearman’s correlation coefficient was calculated for the corresponding data with the null hypothesis that there is no correlation between radial offset magnitudes and distance from the MRI isocenter. Results revealed that there is a statistically significant positive correlation (rho = 0.49, p = 0.01) between the two variables, verifying that distortion levels increase significantly with increasing distance from the MRI isocenter.

Detected MRI distortion magnitudes are reported separately for each axis in Table 4. Maximum and median distortion magnitudes are given. It is noted that distortion is mainly exhibited on the y- (read gradient direction) and z- axes.

**Table 4:** Median and maximum absolute spatial offsets, |*d*_*i*_|, for all 26 control points considered in this study. The MRI DICOM coordinate system is adopted.

	*d*_*x*_(mm)		*d*_*y*_(mm)		*d*_*z*_(mm)		*d*_*r*_(mm)	
	Median	Max	Median	Max	Median	Max	Median	Max
**MRI (forward)**	0.16	1.1	0.45	1.4	0.44	1.6	0.76	1.6
**MRI/CT**	0.41	1.2	0.45	1.4	0.44	1.1	0.92	1.8
**MRI-corrected**	0.14	0.8	0.34	0.8	0.33	1.0	0.51	1.3

### 3.3 MRI/CT overall spatial accuracy

Fig 2b shows the corresponding offset vectors for the MRI/CT co-registered dataset. It is noted that detected offset is associated with the impact of the MRI distortion on the MRI/CT co-registration accuracy. Statistics on the detected offsets are given in Table 4, separately for each axis.

Fig 3 also depicts the correlation between distance from the MRI isocenter and detected radial offset for the MRI/CT dataset. In accordance with the MRI forward dataset, a statistically significant positive correlation is revealed (rho = 0.53, p<0.01), using Spearman’s correlation coefficient. This finding highlights the impact of MRI distortion on the overall accuracy in CT-based SRS/SRT treatment planning workflows.

### 3.4 MRI distortion correction efficacy

Efficacy of the mean image distortion correction technique in 3T T2WIs is shown in Fig 2c. Residual distortion is still noticeable, although considerably reduced as compared with Fig 2a and 2b.

Table 4 allows for a more quantitative comparison between the original (forward) MRI dataset and the corrected one. Detected spatial offsets are reduced as reflected in both median and maximum values reported in Table 4.

Again, a statistically significant positive correlation between increasing distance from the MRI isocenter and detected radial offset is revealed using Spearman’s correlation coefficient (rho = 0.41, p = 0.038). Relevant data are shown in Fig 3. This finding implies that residual distortion, following implementation of the mean image distortion correction, is still position-dependent. Thus, gradient field non-linearities (which are not corrected for by the mean image technique and increase with the distance from the MRI isocenter) are dominant and cannot be considered negligible.

## 4. Discussion

A number of published studies have quantified MRI distortion in high-field systems. Huang et al [41] developed a phantom and a methodology for distortion detection at 3T, although not focusing on SRS/SRT applications. They reported distortion above 1 mm for distances within 18 cm from the isocenter for T1WIs acquired with a receiver bandwidth of 480Hz/pixel. Schmidt et al [24] also performed a phantom-based analysis on T1WIs and concluded that distortion can be limited to <1 mm if higher receiver bandwidths are considered (>800 Hz/pixel). Wang et al [35] implemented the field mapping technique directly to patients to determine susceptibility related distortion levels in T1-weighted imaging. Displacements above 2 mm were detected, although the results refer to a bandwidth of 180 Hz/pixel. Distortion levels in MRI scans are expected to induce further spatial inaccuracies, if an MRI/CT co-registration step is involved in the treatment planning workflow. This parameter was not included in the previously published analyses. In addition, T2WIs were not specifically investigated, although they are routinely employed in SRS/SRT treatment planning in several cases such as vestibular schwannomas [42], trigeminal neuralgia [6] and meningiomas [7] for target and/or critical organ delineation.

In this work, an experimental methodology was implemented to assess the overall MRI distortion specifically in clinically used T2WIs acquired at 3T. Furthermore, the consequent impact on the accuracy of MRI/CT co-registration procedures was investigated. MRI distortion levels may exceed 1 mm (Fig 2a and Table 4), in accordance with the literature for T1WIs, and found to increase with increasing distance from the MRI isocenter (Fig 3). Thus, 3T distortion levels could not be considered acceptable for SRS/SRT target localization at areas far off the isocenter, suggesting that effort should be made to ensure that the target lies in the vicinity of the MRI isocenter. Regarding the studied MRI/CT dataset, similar spatial offsets and trends were detected (Figs 2b and 3, Table 4). The fact that a statistically significant positive correlation between detected offset and distance from the MRI isocenter was also revealed, underlines the impact of MRI distortion on the accuracy of the spatial co-registration process.

Implementing the mean image distortion correction technique in 3T T2WIs considerably reduced detected offsets (Table 4), suggesting that sequence dependent distortion was minimized. However, residual distortion cannot be considered negligible. Distortion related to gradient field non-linearities is not corrected for and, therefore, it dominates the residual spatial infidelity. Gradient field non-linearities are expected to increase with increasing distance from the MRI isocenter [16]. This can be clearly verified in Fig 3, where a statistically significant positive correlation of residual offset with distance to the MRI isocenter is observed.

A number of constraints of this study are noteworthy and may limit the applicability of the results presented herein. First of all, T1WIs were not assessed. Polymerized (high dose) areas demonstrate increased signal in T1WIs, however, the contrast with respect to surrounding low dose areas may not be adequate for the control point localization method and software tool implemented in this study. Furthermore, this is a phantom-based investigation which cannot fully account for patient-induced distortion, originating from clinically relevant challenges, such as air-bone interfaces. Thus, reported distortion mainly comprises B0 inhomogeneity and gradient field non-linearity. Additional patient-induced susceptibility related distortions can, however, be simulated [43] or directly measured [20, 35], with the latter approach burdening the total scanning time of the patient. Most important, susceptibility phenomena can be reduced by sequence optimization. Increasing the bandwidth is the most obvious approach [21, 22, 24], although SNR loss might be a concern if tiny lesions are involved. In this work, distortion was not evaluated in other clinical T2-weighted sequences and further sequence optimization was not attempted. It is noted that distortion is scanner-, sequence-, field-of-view-, parameter-, orientation- and position- dependent and, therefore, thorough distortion assessment should be performed as part of the commissioning and quality assurance programme of an SRS/SRT treatment workflow. Last, our methodology was based on polymer gels which are not typically available in the clinical setting, while production, handling and processing requires user experience and image processing skills. It is noted, however, that the implemented methodology did not involve polymer gel dosimetry which would require additional expertise.

## 5. Conclusion

A polymer gel-based methodology was implemented for distortion assessment in T2-weighted 3T MRIs, using the sequence parameters used in clinical practice for SRS/SRT treatment planning purposes. The methodology was accompanied by image processing routines for control point localization and the relevant overall uncertainty in distortion detection was estimated to be 0.39 mm.

MRI distortion exceeded 1 mm mainly at the locations lying distant from the MRI isocenter. The overall spatial inaccuracy in a treatment workflow involving MRI/CT co-registration was found to be slightly higher compared to that related to an MRI-only workflow. Implementation of the mean image distortion correction technique resulted in considerable distortion reduction. However, residual distortion cannot be considered negligible, mainly due to increased gradient field non-linearities at the periphery of the imaged volume. For all 3 datasets studied, a statistically significant positive correlation was revealed between the detected spatial offset and the distance from the MRI isocenter. This point suggests that effort should be made to ensure that, if possible, the target and surrounding critical organs lie in the vicinity of the MRI isocenter.

Overall results of this work contribute towards the wider adoption of 3T MRI in cranial SRS/SRT procedures. Although total distortion is higher than that anticipated with 1.5T systems, for areas lying close to the MRI isocenter (<5cm) distortion levels can be considered acceptable (<1mm). Moreover, the presented phantom-based methodology can be employed in commissioning and quality assurance programmes of corresponding treatment workflows.
